# Failure to Diagnose Phaeochromocytoma Preoperatively: A Case Report and Review of Diagnostic Criteria

**DOI:** 10.1155/2011/841510

**Published:** 2012-01-18

**Authors:** Ileana Antonopoulou, Mark Heining

**Affiliations:** ^1^Department of Anaesthesia, Nottingham University Hospitals, Hucknall Road, Nottingham NG5 1PB, UK; ^2^Department of Anaesthesia, Nottingham City Hospital, Hucknall Road, Nottingham NG5 1PB, UK

## Abstract

We present a case in which phaeochromocytoma was not diagnosed preoperatively despite extensive investigation. There was significant haemodynamic instability during surgery. We review current diagnostic criteria with a view to reducing such a risk in future.

## 1. Introduction

Phaeochromocytoma is a rare catecholamine-secreting tumour arising from chromaffin tissue. It may precipitate life-threatening hypertensive crises or cardiac arrhythmias and so preoperative diagnosis is essential. Mortality associated with surgery in an undiagnosed phaeochromocytoma is said to be up to 80% [1]. We present a case of a known adrenal tumour in which standard preoperative tests failed to diagnose phaeochromocytoma. We discuss the range of available diagnostic tests and make recommendations for their possible applications.

## 2. Case Report

A 54-year-old female presented for laparoscopic adrenalectomy, with a diagnosis of a vascular nonfunctioning adrenal tumour.

She had originally presented about 18 months previously with an episode of abdominal pain, dizziness, and vomiting. Blood pressure on admission to hospital was normal, although it was noted in the referral letter from her family doctor to have been 240/60 mmHg with a heart rate of 90 beats per minute.

 Her other medical histories included hypercholesterolaemia, polycystic ovarian syndrome, and morbid obesity treated with gastric banding 8 months before the current admission: this had resulted in a weight reduction from 117 kg (Body Mass Index, BMI 46.3) to 64.5 kg (BMI 25). She had diabetes mellitus, which had previously been controlled with insulin but since her bariatric surgery had been controlled with metformin. Her other drug treatment comprised fenofibrate, citalopram, vitamin B_12_, folic acid, and multivitamins.

 Following abdominal ultrasound on admission, she underwent abdominal CT on the same day, followed by a succession of other imaging modalities because of diagnostic uncertainty. She also had biochemical tests for phaeochromocytoma, Cushing's syndrome, and Conn's syndrome. Results of imaging and diagnostic tests are summarised in Table 1.

 The anaesthetic technique for the laparoscopic adrenalectomy consisted of induction with midazolam 2 mg, alfentanil 1 mg and propofol 100 mg, neuromuscular blockade with rocuronium 50 mg, and maintenance of anaesthesia with oxygen/air/sevoflurane. Fentanyl was given to a total dose of 400 micrograms, and further rocuronium titrated against the response to a peripheral nerve stimulator. Blood pressure monitoring was with an automatic noninvasive technique, and no central venous pressure monitoring was used.

 During mobilisation of the tumour, blood pressure rose to 266/124 mmHg. Initially this was treated with increased inspired concentration of sevoflurane and increments of fentanyl. Similar episodes occurred on two further occasions, and an esmolol infusion was given during these episodes (total dose 200 mg). Following removal of the tumour which took approximately three hours altogether, there was no further recurrence of hypertension and no hypotension. The patient was extubated at the end of the procedure and returned to a general surgical ward. She remained well until her discharge from hospital two days later.

 Histology of the adrenal gland, reported six days later, showed that about half of it consisted of a distinct greyish-red lesion. Histology of this lesion showed an arrangement of small cells with hyaline globules, together with sustentacular cells. This is characteristic of a phaeochromocytoma and is known as a *zellballen.* The diagnosis was made by a pathologist experienced in the histology of adrenal glands.

## 3. Discussion

### 3.1. The Case

This patient presented for surgery with a diagnosis of nonfunctioning adrenal adenoma. Phaeochromocytoma, Cushing's syndrome, and Conn's syndrome had been ruled out by appropriate diagnostic tests, some of which had been repeated on several occasions over several months of tumour surveillance.

 During mobilisation of the adrenal mass, she became very hypertensive. The cause of this was not immediately obvious, especially since we were working on the assumption that phaeochromocytoma was not a possible diagnosis. Persistence and recurrence of hypertension appeared to rule out equipment malfunction, so our first response was to increase the inspired concentration of volatile agent and give increments of opioid.

 Recurrence of hypertension despite these manoeuvres prompted us to start an infusion of esmolol. With hindsight this is not a logical choice since beta-blockade can worsen hypertension in the presence of a phaeochromocytoma, by opposing beta-adrenoceptor-activated vasodilatation in skeletal muscle beds. However, we were working on the assumption that this was definitely not a phaeochromocytoma and the priority was myocardial and cerebral protection in the face of severe hypertension. In the time it took to reconsider the management, surgery was completed and the blood pressure had returned to normal.

 At the time we were not aware of the limited sensitivity of 24-hour urine assays in the diagnosis of phaeochromocytoma. If we had been, we might have had a higher index of suspicion and managed the hypertension with alpha-blockade (e.g., phentolamine) or other vasodilator (e.g., sodium nitroprusside) [2].

### 3.2. Diagnostic Tests

The choice of diagnostic test is based on the index of suspicion of phaeochromocytoma. Plasma metanephrine has the highest sensitivity (96%) but lower specificity (85%). In comparison, 24-hour urinary catecholamines and metanephrines have a sensitivity of 87.5% and a specificity of 99.7% [3]. The international consensus on screening recommends 24-hour urinary catecholamines with or without urinary metanephrines as the standard screening tool [4]. The diagnosis should be considered in patients (especially young patients) whose hypertension is difficult to control, or in whom there are other characteristic symptoms, such as headache, palpitations, and sweating [5, 6]. There are some groups of patients in whom the index of suspicion is higher, and these include those with persistent hypertension, familial endocrine neoplasia syndromes, or suspicious appearances on imaging [7]. It may then be appropriate to also measure plasma free metanephrines [8, 9].

 Our patient had none of the characteristic features of phaeochromocytoma, instead presenting acutely with haemorrhage into the adrenal gland. She had an isolated blood pressure reading in the hypertensive range, and this was when she was in acute pain at her initial presentation. It is not unknown for patients with phaeochromocytoma to be normotensive [10], but safe management of these patients still requires treatment with alpha-blocking agents preoperatively. We believed that phaeochromocytoma had been excluded in our patient by the finding of 3 normal 24-hour urinary catecholamines and metanephrines [11, 12]. We were not aware at that time that the usual guidelines do not apply to asymptomatic patients; otherwise we might have requested plasma metanephrine assay as well.

 The only suspicious finding, which might have prompted further investigation, was the report of slightly atypical features on the MRI scan. We were thus presented with a normotensive patient who had had a diagnostic test within the normal range on 3 occasions. It was difficult to justify further investigation.

**Further Comment**

During the process of obtaining written consent from the patient for publications of this case, she told us that she had actually had symptoms of headache, dizziness, and palpitations and had also self-measured her blood pressure in the hypertensive range.

## 4. Conclusion

Negative urinary catecholamines in a histologically or imaging-confirmed phaeochromocytoma are not uncommon [10], but here we present a case of failure of both biochemical and imaging detection with obvious consequences. We present this case in order to alert anaesthetists to the limitations of diagnostic tests for phaeochromocytoma. Urinary catecholamines may be a useful screening tool in symptomatic patients, but if there is any doubt at all (e.g., atypical imaging appearance), then further biochemical tests such as plasma metanephrines should be done. It may also be advisable to take a very careful history in case patients are not forthcoming with symptoms.

##  Conflict of Interests

The authors declare that there were no external funding and no conflect of interests. 

## Figures and Tables

**Table 1:** Diagnostic tests performed.

Imaging studies			
Modality	Timing	Result	
Ultrasound scan	Day 0	5 × 4 cm mass upper pole of left kidney	
CT scan	Day 0	5 × 5.7 cm L retroperitoneal mass suggestive of haemorrhage into adrenal cyst	
CT scan	At 2 months	Decrease in size to 3.2 × 3.1 cm suggestive of adrenal vascular tumour	
CT angiogram	At 2 months	Vascular L adrenal tumour	
CT angiogram	At 6 months	Further decrease in size to 3 × 2.5 cm, with the appearance of a vascular adrenal tumour	
MRI	At 8 months	Adrenal mass 2.9 × 2.1 cm. Not a simple adenoma	

Biochemical studies			
Test	Timing	Result	Normal range

Random serum cortisol (mmol · L^−1^)	Day 1	489	140–690
Random serum cortisol (mmol · L^−1^)	Day 1 (repeat)	362	140–690
Serum Aldosterone (pmol · L^−1^)	Day 1	129	111–860
Serum Renin (mU · L^−1^)	Day 1	20	8.3–46.3
*24-hour urinary catecholamines*			
Noradrenaline (nmol/24-hour)	At 2 months	266	0–430
Adrenaline (nmol/24-hour)	At 2 months	41	0–70
Dopamine (nmol/24-hour)	At 2 months	1690	0–2700 n
*24-hour urinary metanephrines*			
Normetadrenaline (nmol/24-hour)	At 2 months	2972	0–4900
Metadrenaline (nmol/24-hour)	At 2 months	1293	0–2000
*24-hour urinary catecholamines*			
Noradrenaline (nmol/24-hour)	At 9 months	313	0–430
Adrenaline (nmol/24-hour)	At 9 months	53	0–70
Dopamine (nmol/24-hour)	At 9 months	1480	0–2700
*24-hour urinary catecholamines*			
Noradrenaline (nmol/24-hour)	At 18 months	133	0–430
Adrenaline (nmol/24-hour)	At 18 months	35	0–70
Dopamine (nmol/24-hour)	At 18 months	783	0–2700

24-hour urinary catecholamines collected on the day of surgery following the hypertensive crisis were also normal.
